# Nanomaterials: Solutions to Water-Concomitant Challenges

**DOI:** 10.3390/membranes9030040

**Published:** 2019-03-14

**Authors:** Shaik Anwar Ahamed Nabeela Nasreen, Subramanian Sundarrajan, Syed Abdulrahim Syed Nizar, Seeram Ramakrishna

**Affiliations:** 1 Department of Mechanical Engineering, National University of Singapore, 2 Engineering Drive 3, Singapore 117581, Singapore; mpesaan@nus.edu.sg (S.A.A.N.N.); sundar@nus.edu.sg (S.S.); 2 NUS Nanoscience and Nanotechnology Institute, National University of Singapore, 2 Engineering Drive 3, Singapore 117581, Singapore; nnisasn@nus.edu.sg

**Keywords:** pollutant, electrospinning, nanofiber membranes, metal oxides, carbonaceous materials, water filtration

## Abstract

Plenty of fresh water resources are still inaccessible for human use. Calamities such as pollution, climate change, and global warming pose serious threats to the fresh water system. Although many naturally and synthetically grown materials have been taken up to resolve these issues, there is still plenty of room for enhancements in technology and material perspectives to maximize resources and to minimize harm. Considering the challenges related to the purification of water, materials in the form of nanofiber membranes and nanomaterials have made tremendous contributions to water purification and filtration. Nanofiber membranes made of synthetic polymer nanofibers, ceramic membranes etc., metal oxides in various morphologies, and carbonaceous materials were explored in relation to waste removal from water. In this review, we have discussed a few key materials that have shown effectiveness in removing pollutants from waste water, enabling solutions to existing problems in obtaining clean drinking water.

## 1. Introduction

Indicators of the United Nations Water statistics shows that the Earth’s entire water capacity is 1.4 billion km^3^. About 2.5% of the total volume is accounted for by fresh water, which totals about 35 million km^3^ in volume, with the remaining water resource existing as saline water. Snow and ice in the Arctic and Antarctic cover 70% of the available fresh water in mountainous regions (24 million km^3^). Shallow and deep ground water basins lock up approximately 30% of the remaining water, which is up to 2 km^3^. Only 0.3% accounts for rivers and lakes, for a total volume of 105,000 km^3^ [1]. Overall, from the 97% available fresh water, about 70% is used for irrigation, 22% for industrial use, and 8% for domestic purposes. The world’s seven billion people consume about 54% of the accessible fresh water from underground aquifers, rivers, and lakes, and the remaining 46% left unused. Almost 97% of global water resources are from seas and oceans. Though we have enough water resources, many potential hazards such as global warming due to overpopulation, urbanization, and industrial growth affect the availability of water resources. Based on the demands of population growth, many sectors are expanding, imposing excessive demands on water, leading to a much-expected depletion of water and its resources.

Industrialization and urbanization accumulate pollutants such as heavy metal ions [2], dyes [3], pharmaceutical wastes [4], etc. in water. Toxic heavy metals like Cd, As, Hg, and Pb in natural potable water exceed the permissible tolerance limit. Humans contact with such heavy metals poses a major health risk, due to its carcinogenic and toxic effects [5]. In recent years, the removal of heavy metals has been an extensive research topic. These heavy metals are not only causing pollution to water, but they are also life-threatening [6]. Some of the most toxic and carcinogenic heavy metals include Pb(II) [7], Cd(II) [8], As(II) [9], Cu(II) [10], Ni(II) [11], As(III) [12], and Cr(VI) [13] and so on. Some of the metal cations can even cause damage to the central nervous system and kidneys in humans.

Another pollutant which is the most life threatening to human is dyes. Textile industries are the foremost source of dye pollutants in water contamination. Dye removal is an extremely important issue to deal with, as it is well-understood that it causes serious harm to humans. Some of the key consequences of dyes include the inhibition of light to penetrate into water, which in turn hampers photosynthesis in aquatic plants. Carcinogenic effects have also been observed in a few dyes, and their degraded products are present in surface water [14]. Hence, it is important to clean or to pretreat these dye effluents before discharging them into any water resources. Reports from the Odour Index, managed by SDC (Society of Dyers and Colorists) shows there are about 100,000 different types of dyes that are synthesized and commercially available [15]. An estimated 700,000 tonnes of dyes are produced worldwide. About 2.4% of this total run off as industrial waste water after use; it is difficult to treat organic dyes through normal procedures, and it is highly recommended that they be degraded by chemical methods. The majority of dyes are dangerous, e.g., methyl red is a mono azo dye that is used in textiles and in other commercial products, which poses serious damage to the digestive tract, causing irritation if it is inhaled or swallowed, as well as skin and pharyngeal sensitization, and so on [16]. Microbes in the water, as pathogens, can pose a public health hazard, leading to severe gastrointestinal illnesses. Coagulation [17], flocculation [18], biological oxidation [19], activated carbon [20], and chemical precipitation [21] adsorption are a few common methods that are available for color removal [22]. Similarly, oil spills in various forms are also a cause for water pollution. Due to many modern techniques, oil spills [23] from industries and bilge water [24] increase the level of contamination in water resources. It is equally important to remove oil, which affects the food chain if it persists in the long-term.

It is well-understood that microbes such as bacteria, viruses, and other biological entities contaminate the air, water, and soil, and thereby create serious harmful effects to humans and other living things. With an increasing population, awareness of public health is needed, due to the pathogenic effects caused by microorganisms. This leads to an increase in the requirement for antibacterial materials in medicines, food, etc. Millions of people in developing countries die due to the non-accessibility of pathogen-free drinking water. Even chlorination, which is used to decontaminate water, will also react with naturally-occurring organic materials in water and produce by-products that are highly hazardous. Human infections by certain bacteria and protozoan parasites are generally a result of contaminated water. *Shigella* spp., *Vibrio cholera*, *Escherichia coli*, *Salmonella* spp., and *Campylobacter* spp. are a few major pathogenic bacteria that are of concern in water. Waterborne protozoan pathogens include *Naegleria fowleriand*, *Cryptosporidium parvum*, *Giardia duodenalis*, *Entamoeba histolytic*, and *Acanthamoeba* spp.

Many new technologies have been established, but the majority are trial-and-error systems that forth potential and effective systems for desalination and pollutant removal. There are many scientific methods and materials that have been proposed and tested for the purification of water samples. Among those materials, nanomaterials in the form of nanoparticles or nanofibers with different morphologies have potential impacts for treating water, and have been observed to be very effective in removing pathogens, microbes, heavy metal ions, toxic metals, dye, oil, and other harmful organic solvents. In this review, we have discussed a few nanomaterials, such as the role of nanofiber membranes, in the effective removal of heavy metal ions, dyes, and oils. This discussion is further extended to study the antibacterial activities of such materials. Metal oxides and carbonaceous particles with high efficiency towards water purification are also discussed briefly.

## 2. The Role of Nanomaterials for Effective Pollutant Removal—Heavy Metals, Dyes, and Antibacterial Activity

### 2.1. Nanofiber Membranes as Pollutant Removal Media

Nanofiber membranes are commonly studied materials for water filtration applications [25]. Many reviews on filtration using nanofiber membranes have been reported earlier by Ramalingam et al. [26] on air filtration, and by Shaik et al. [27] on water filtration. Polymer nanofibers that are fabricated by the electrospinning method have been shown to have unique properties in filtration studies.

The removal of microparticles by using polysulphone (PSu) nanofibers spun onto the backing material have been investigated by Renuga et al. [28]. High flux-prefilters with a maximum capability of loading were tested, due to the high porosity and surface area of the nanofiber membrane. The membrane had the ability to eradicate 99% of 10^−7^ µm sized particles without any permanent fouling. With lower sized particles, it forms a cake, and hence this membrane was suggested for use as a prefilter for various microparticles. Similarly, high-flux microfiltration (MF) filters were made by using polyvinyl alcohol (PVA) polymers electrospun onto a microfibrous support, and crosslinked with glutaraldehyde in acetone. The reduction in the mean pore size from 0.3 µm to 0.2 µm enhanced the MF efficiency to about 3–7 times for nanofiber membranes when compared to the commercial membrane (Millipore) [29]. Also, an increase in the permeation flux improved the particle removal quite efficiently. Hongyang et al. [30] reported the synthesis of an ultrafine cellulose membrane of about 5–10 µm diameter, with a high surface volume and good chemical resistance. It showed an improved flux of about 2–3 times, compared to commercial membranes (gs9035 Millipore). Shaik et al. [31] reported a slight modification in the base polymer of an electrospun nanofiber membrane (ENM) that improved permeation flux. The hydrophilic poly (vinylidinefluoride)-hydroxyethyl methacrylate (PVDF-HEMA) membrane, developed by in situ polymerization of HEMA with PVDF, exhibited a high flux rate. A thin film coating was applied onto the electrospun membranes to reduce the pore size, which improved the flux to 60%. It was concluded that hydrogen bonding and in-situ polymerization resulted in the hydrophilic nature and high permeation flux of the polymer after spinning and lowering of the pore size.

**Figure 1:**
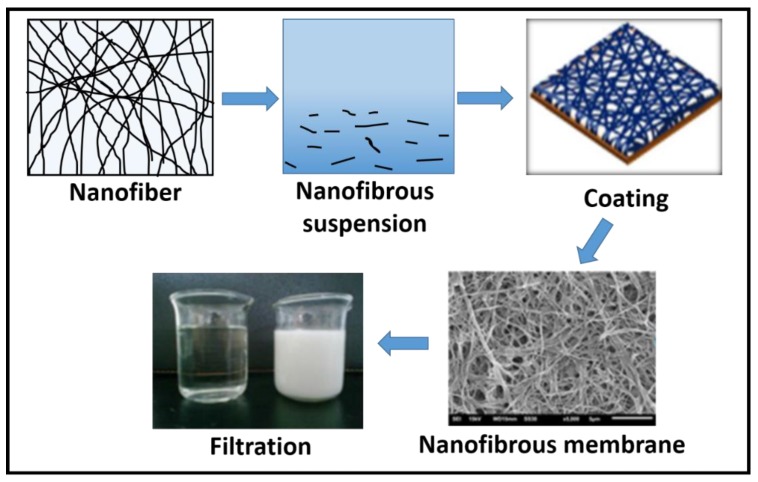
Nanofiber preparation and separation of the TiO_2_ suspension Adopted from Ref. [32].

Further, enhancements in the flux were studied by Mufang et al. [32] using hydrophilic polyvinyl alcohol-co-ethylene. The membranes were used as filter media for many of the nanoparticles, e.g., 99% of the nanoparticles were removed from the TiO_2_ suspension, and they exhibited high flux rates, and were superior to other MF membranes (Figure 1).

#### 2.1.1. Heavy Metal Removal

Heavy metal ions are one among the major threats to water resources, and many efforts has been made to eliminate toxic heavy metals from water. Adsorption, surface reactions, or catalytic reduction are a few possible methods that are used to remove toxic particles. For example, alumina and micellar-enhanced filtration (MEF) adsorption are used to remove Cu(II) ions in ground water [33]. Sodium dodecyl benzene sulfonate (SDS) micelles help with the electrostatic removal of cations. These micelles exhibit as secondary contamination when they are filtered through a chloridized polyvinyl chloride (PVC) nanofiber membrane. Alumina particles were employed to cure this problem, as the negatively charged micelles were attached to positively charged alumina particles, and they removed 100% Cu(II) ions from groundwater. Chromium, a heavy metal that is highly toxic in water was studied by Jianqiang et al. [34]. In his study, an electrospinning method was applied to produce a polyacrylonitrile/polypyrrole (PAN/PPy) core/shell nanofiber sheet, and this was subjected to chemical oxidation. Selective adsorption of anions due to the interaction between PPy and heavy metal anions during adsorption was the key process for the model. An anion exchange process between Cr(VI) and Cl^−^, followed by a redox process with Cr(VI) ions, was detected. The adsorption relied on the time taken for protonation of the PAN/PPy nanofiber sheet and it increased with increase in protonation time. It showed good selectivity for Cr(VI) reduction to Cr(III), and other anions (SO_4_^2−^, Cl^−^, and NO^3−^), except for PO_4_^3−^ which remained unaffected. Other heavy metal ions such as Fe(III), Cd(II), and Cu(II) were removed using amide oxime-modified PAN (AOPAN)/cellulose acetate (CA), via the electrospinning technique [35]. The saturation absorption of the nanofibrous membrane for these metal ions was found to be 7.47, 1.13, and 4.26 mmol/g. Excellent reproducibility with over 80% adsorption and desorption capacities were observed with the modified membrane. A zeolitic imidazolate framework (ZIF-8)-coordinated electrospun nanofiber membrane was tested with heavy metal removal by Want et al. [36]. The property of a high surface area of the electrospun PAN membrane, along with properties of the microporous ZIF-8 material as active sites, supports the effective removal of heavy metals, resulting in a high permeation flux (180 L/m^2^/h/psi). This comparison shows that the coordinated PAN membrane showed thrice the heavy metal removal capacity than the normal PAN membrane.

Aerogels formed by a quaternary ammonium-functionalized cellulose nanofiber was studied by Xu et al. [37] for Cr removal. A large surface area of fibers triggers the speedy and active rejection of Cr(VI) from contaminated water. Over 99% of Cr(VI) was removed in 50 min with 1 g of aerogel, in 1 L of 1 mg/L solution. Xiao et al. [38] reported a nanofiltration membrane formed via the phase inversion method, after cross-linking amines with 1% trimesoylchloride by interfacial polymerization (IP). The membrane had a smaller pore size, allowing the rejection of salt (NaCl) from 74% (without IP) to 91% (with IP). Kyunghwan et al. [39] reported on electrospun PAN as a midlayer support in thin film nanocomposite (TFNC) membranes for nanofiltration. Different ratios of bipiperidine and piperazine formed the top polyamide barrier layer. Salt rejection (MgSO_4_) was tested with these membranes, and it was found that the nanofiber membrane showed 2.4 times more flux than a thin-film composite membrane (TFC). Poly(methyl methacrylate) (PMMA) nanofibrous membranes doped with rhodanine (2-thioxo-4-thiazolidinone) were employed to remove heavy metals such as silver and lead [40]. The immobilization of carbazol-based Schiff base organic molecules into a polyvinyl alcohol (PVA)–tetraethyl orthosilicate (TEOS) inorganic polymeric support was reported by Arash et al. [41] to remove highly toxic mercury (Hg(II)). The composite detects Hg(II) in the range of 0.020–0.50 ng/mL, with a limit of detection (LOD) to 0.0180 ng/mL and a removal efficiency of 97–99%. In another study, cysteine-modified polymer nanofibers were employed in Tannery waste water samples for the removal of Cr(III) [42]. At pH 5.5, almost 99% of Cr(III) was removed with only 0.1 mg of nanofibers per mL of tannery waste water. About 1.75 g Cr/g of polymeric material was concluded to deliver the maximum removal capacity. A high surface-to-volume ratio, and large numbers of cysteine groups with binding affinities to heavy metals, are a significant cause of the high metal-removal capacity. Heavy metal ions such as Cr(VI), Co(III), and Cu(II) were efficiently removed with good stability and reproducibility, using a chitosan-based nanofiber membrane [43]. Modification of the chitosan surface as a rich amino-functionalized chitosan membrane by grafting of polyglycidyl methacrylate (PGMA) and polyethylenimine (PEI) effectively improves the removal ability of the ENM. The adsorption process follows a pseudo-second-order model with maximum adsorption capacities of 138.96, 68.31m and 69.27 mg/g respectively. Recently, polyaniline-grafted chitosan (PGC) was reported as a sorbent material for the removal of Pb(II) and Cd(II) ions. Monolayer Langmuir isotherm adsorption was recorded to be 16.07 mg/g and 14.33 mg/g for Pb(II) and Cd(II) ions, respectively [44]. Similarly, a surface-modified polytetrafluoroethylene (PTFE) membrane using a hyper-branched poly- amidoamine (HPAMAM) showed good efficiency towards the removal of Cu^2+^ ion. The water flux improved, and the rejection property was undisturbed when using the modified PTFE membrane. Pure PTFE showed 0 L/m^2^/hr flux, whereas HPAMAM grafted PTFE membranes showed 635 ± 9 L/m^2^/hr flux. Approximately 1.42 g/m^2^ Cu^2+^ ions were adsorbed by the grafted polymer from an aqueous solution, with a desorption rate of 90% under index acidic conditions [45]. A few more reports on composite nanofiber mats are highlighted in Table 1.

#### 2.1.2. Antibacterial Activity

Hongyang et al. [54] reported a MF membrane produced using surface-functionalized PAN nanofibers. The polymerization of dual- and tri-vinyl monomers on the surface of PAN nanofibers improved the membrane’s mechanical strength, due to cross-linking. Efficient removal of bacteria and viruses were observed, with 99.99% retention of bacteriophages and 99.9999% retention of bacteria. Moreover, these membranes exerted high permeation flux and low rates of pressure drop. Doping of a poly(ether sulfone) (PES) ultrafiltration membrane comprising halloysite nanotubes with copper ions acts as a good anti-bacterial agent [55]. The modified membrane showed high hydrophilicity and high water flux. Antibacterial activity with *E. coli* and *S. aureus* were tested, and it shows excellent properties against these microorganisms.

Polylactic co-glycolic acid (PLGA), a biocompatible polymer nanofiber loaded with different concentrations of silver nanoparticles (0, 1, 7 wt %) and evaluated for liver cancer cell lines. Increased concentrations of silver nanoparticles (Ag NPs) showed a remarkable improvement in polymer anticancer properties. PLGA nano-fibers with 1% of nanosilver loading showed 8.8% of anticancer activity, and by increasing the concentration to 7%, the anticancer activity was enhanced to 67.6%. Further, these nanofiber materials were extended to understand the antibacterial activities against five bacterial strains, namely; *E. coli*, *S. aureus*, *Bacillus cereus*, *Listeria monocyte* genes, and *Salmonella typhimurium*, by using the disc diffusion method, and they showed enhanced inhibitory effects. In the case of 7% PLGA/AgNPs, the loaded sample inhibited all strains, whereas for only one strain (*B. cereus*), inhibition was observed for a 1% PLGA/AgNP-loaded nanofiber membrane [56]. The characteristics of the filter mat showed excellent behavior when composites such as silver-infused TiO_2_/nylon-6 were involved [57]. Silver or AgNPs were photocatalytically reduced under UV irradiation in TiO_2_/nylon 6 nanofiber mats. Spider wave-like structures caused by TiO_2_ NPs in the nylon 6 solution facilitated the reduction of AgNO_3_ to Ag, and the mats exhibited good antibacterial activity against *E. coli*. The reuse of nanoparticles will make the material an economically friendly catalyst and filter media.

The colonization of bacteria on titanium-implanted Sprague-Dawley rats was examined by Samuel et al. [58], using antibacterial nanofiber membranes. Nanofiber membranes loaded with drugs such as biocides (fusidic acid and rifampicin) were implanted onto the titanium surface. Upon injection, *S. aureus* the antibacterial nanofiber membranes prevented the adhesion of bacteria onto the implanted site. Similarly, Daels et al. [59] calculated the antibacterial properties of polyamide membranes loaded with five different biocides, to treat hospital waste water. About 4.0 log_10_ reductions of bacterial numbers were observed for drug-loaded membranes, which was higher than the control (5.2 log_10_ reduction). Another report on surface-modified nanofiber membranes, showing highly effective antibacterial activity against *E. coli* and *S. aureus* activity, was reported by Chen et al. [60]. Plasma pretreatment, UV-induced grafting of 4-vinylpyridine, followed by the quarternization of grafted pyridine groups was conducted on polyurethane nanofiber membranes. Poly(hexamethylene guinidine hydrochloride) (PHGH) was attached to flexible spacers, which extended from amino groups from the reduced nitrile groups on PAN nanofibers, were produced to study the antibacterial activity against *S. aureus* and *E. coli*. The surface-modified nanofiber mats showed ease of cleaning, due to the hydrophilic spacers, and high antibacterial activity, even after a few cycles of antibacterial assays [61].

#### 2.1.3. Dye Removal

Functional group-modified PAN using sodium hydroxide and bicarbonate act as a sorbent for malachite green (MG) and cationic dyes. A modified PAN membrane removed 99% of MG, with an adsorption capacity of 200 mg/g (five times higher than non-functionalized PAN) [62]. Composite nanofibers made of PAN–polyamidoamine (PAMAM) were synthesized and investigated with Direct Red 23 dyes and Direct Red 80, with a dye adsorption capacity amounting to 2000 mg/g and 1666.66 mg/g, respectively. The increase in dye adsorption was due to higher loading of the active polymer, which created more active sites for dye adsorption on the surfaces of the fibers [63]. A PVA/CA composite was reported for the removal of MG, by Xiaoli et al. [64]. The foam exhibits well-interconnected pores and good mass transfer properties, and it removes dye even at very low concentration (<100 mg/L), and it is reusable. A membrane filter made from nylon-6 nanofibers on a carbon-coated polyurethane substrate was tested by using C.I. Direct yellow 12. A filtration efficiency of 98% was achieved in 150 ppm of coagulant material at 0.75 bar pressure [65]. The application of a fast molecular filtration method, was reported to remove the hydrophilic dyes using poly(ether amine) (PEA) nanofiber membranes. UV dimerization of the coumarin groups were cross-linked to the PEA membrane for adsorption studies. Strong adsorption with Ponceau S (PS), Ponceau SX (PSX), Orange G (OG), Rose Bengal (RB), and Bismarck brown Y (BY), were observed, and weak adsorption to rhodamine 6G (R6G) and Methylene Blue trihydrate (MB), was reported. These membranes also separate mixtures of PS/MB in aqueous solution through molecular filtration, with a very high flux rate of 2870 L/m^2^/hr [66]. Another composite nanofibrous filter medium of polyamide 6/chitosan was fabricated onto a satin fabric substrate by electrospinning. Polar Yellow GN and Solophenyl Red 3BL, and acidic and direct dyes were investigated at different pH levels and initial dye concentrations, along with different membrane parameters. A 95% removal rate of Polar Yellow GN, and a 96% removal rate of Solophenyl Red 3BL was observed when using these mixed nanofiber membranes [67].

Nanoparticle-loaded nanofiber membranes also exhibit good dye-removing capabilities. For example, AgNPs loaded on electrospun TiO_2_ nanofibers for dye degradation were studied by Lei et al. [68]. Under solar irradiation, the Ag/TiO_2_ nanofiber membrane achieved 80% dye degradation under within 30 min, due to high permeate flux when compared to a commercial P25 membrane. Similarly, an organically modified montmorillonite composite with PAN membrane coated with TiO_2_ improved the adsorption of methylene blue (MB) [69]. Another report on the removal of MB was documented by Aluigi et al. [70] by using a keratin nanofibrous membrane (dia. 220 nm), according to batch adsorption tests. Functionalized PES nanofibrous membranes were synthesized by Xu et al. by blending PES with a copolymer of acrylic acid and methyl acrylate [71]. High porosity, large specific area, and abundant carboxyl groups showed the largest adsorption capacity, removing 2257.88 mg/g of methylene blue dyes. Its recyclability of up to five cycles, with a competence of 81.5%, a purification efficiency of >99% and at a high flux of 100 mL/min are other key promising properties. Poly (L-lactic acid) (PLLA) nanofibrous adsorbents produced through air plasma etching treatment effectively removed Methylene Blue dye via electrostatic interaction. The presence of active binding sites after plasma etching, and the high surface area of the membrane were factors contributing to its performance. Its adsorption kinetics fit well with a pseudo-second-order model, revealing a monolayer for chemical adsorption, and it also exhibited good recycling efficiency [72]. Hydrolysis of a polymer of intrinsic microporosity (PIM-1) by 65% to 99%, with a fiber diameter ranging from 0.58 to 1.21 μm, showed extensively high performance in the removal of organic dyes from the wastewater [73]. With the concertation of 20 mg/L dye solution, PIM-1 nanofibers exhibited a removal efficiency of about 157 mg/g for Methylene Blue, with a maximum dye removal efficiency of 272 mg/g, and also of 4 mg/g for Congo Red. The modification of PAN nanofibers with chelating agents such as ethylenediaminetetraacetic acid (EDTA) and ethylenediamine (EDA) changed the distribution of nanofibers, resulting in reduced softness but with no effect on the structure. Their efficient sorption of Methyl Orange (MO) and Reactive Red (RR) showed maximum absorption capacities of 99.15 mg/g and 110 mg/g, respectively [74].

### 2.2. Metal Oxides as Pollutant Removal Media

The macroscopic properties of metal oxides vary with the intrinsic insertion of nano-sized particles. These nanostructures enhance many properties of the individual particles, which could lead to major applications in many desired fields. Particles with defined shapes, and uniform domains and morphologies possess unique properties, which differ from their macroscopic properties. Many such nano-metal oxides have been discussed under these topics, for the effective removal of heavy metals and dyes, and as materials for the study of antibacterial activity against bacteria and viruses.

#### 2.2.1. Heavy Metal Removal

**Scheme 1:**
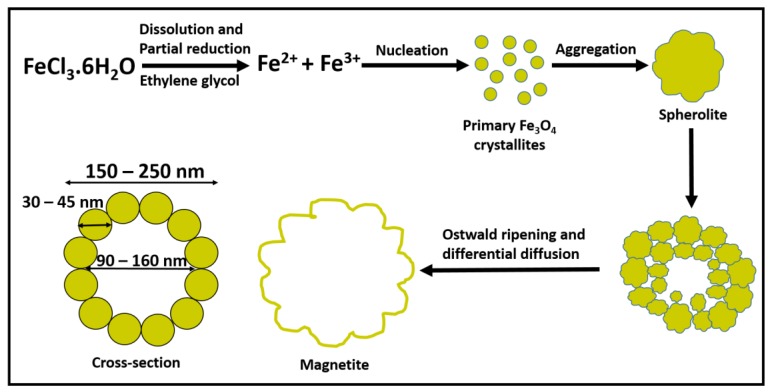
Schematic of magnetite nanosphere formation and evolution. Adopted from Ref. [76].

Various techniques have been employed to deal with the removal of heavy metals—chemical precipitation, adsorption, and electrochemical methods. In most of these techniques, the adsorption method is the most logical and easiest method for removal studies. Although many techniques are available, it is the material that matters in the efficient removal of pollutants. Macro- to nano-sized materials play a predominant role in the efficiency of heavy metal removal. Jianming et al. [75] reported core shell-nanostructured Fe_3_O_4_@SiO_2_–NH_2_ nanoparticles for the effective removal of toxic Pb(II) metals (QM = 243.9 mg/g at 25 °C) in which the presence of amine functional groups on the surface is responsible for the majority of the removal mechanism. Mesoporous magnetite nanospheres synthesized using a solvothermal method were reported by Madhu et al. [76]. Their adsorption at mild acidic pH values and at different temperatures enabled the selective removal of Cr^6+^ and Pb^2+^ effectively, and this fitted well with pseudo-second-order kinetics. The Langmuir adsorption capacities were 19 and 9 mg/g for Pb^2+^ and Cr^6+^, respectively, and the spheres were reusable after further isolation (Scheme 1).

Similarly, γ-Fe_2_O_3_ (maghemite) nanoparticles were employed by Ali et al. [77] for the selective removal of toxic metal ions such as Zn(II), Pb(II), and Cd(II). The observed selectivity order for removal was Zn(II) 4.79 mg/g, Pb(II) 10.55 mg/g, and Cd(II) 1.75 mg/g, showing that Pb-Zn waste waters can be decontaminated effectively by using γ-Fe_2_O_3_. Surface-modified Fe_3_O_4_ nanoparticles embedded in silica were mixed with a PES membrane for Cu(II). Due to the improved hydrophilicity and the nucleophilic functional groups on the nanoparticles, the copper removal capability increased remarkably [78].

Bimetallic nanoparticles made of Fe/Ni reinforced by kaolinite (K-Fe/Ni) were used to target Cu(II) and nitrate ions. The removal of both metals and anions were mutually affected when using K-Fe/Ni. A total of 42.5% of nitrate was degraded when Cu(II) was present, and if Cu(II) was reduced to Cu^0^ onto the surface of kaolinite-supported Fe/Ni, it forms a new catalyst, K-Fe/Ni/Cu [79]. For Modified sewage sludge using Fe_2_O_3_ containing a high surface area, the pore volume was applied for the removal of ions such as Ni(II), Cu(II), Pb(II), and Cd(II) [80], and the maximum adsorption was exhibited to be 7.8, 17.3, 14.7, and 42.4 mg/g, respectively, at neutral pH. 3-dimensional organized mesoporous silica (OMS) coated with Fe_2_O_3_ and Al_2_O_3_ was channeled for As(III) and As(V) removal [81]. An 8% metal-coated sample showed optimal conditions for removal efficiency. Maximum adsorption capacity of 55 mg As(V) g^−1^ for 8% Al oxide coated material at pH 5, and 35 mg As(V) g^−1^ for iron oxide-coated material at pH 4 was reported.

#### 2.2.2. Dye Removal using Metal Oxide as a Sorbent

Fe_3_O_4_ loaded with Fe nanoparticles was analyzed for its capability to remove methyl red (MR). It exhibits an excellent absorption of 625 mg/g, which was higher when compared to previously reported zeolite and activated carbon [82]. Hierarchical porous iron oxides produced by a thermal conversion method was proven to be more effective towards Congo red dye. The Langmuir constants for Fe_3_O_4,_ α-Fe_2_O_3_, and γ-Fe_2_O_3_ were found to be 84.96 mg/g, 139.86 mg/g, and 69.35 mg/g respectively, indicating adsorbent behavior of iron oxide superstructures [83].

Fe_2_O_3_-Al_2_O_3_ nanocomposites have a good adsorbent property towards Congo red. The removal capacity for γ Fe_2_O_3_-Al_2_O_3_ phase was recorded as 498 mg/g. Among the three different phase oxides, γ-phase acts as a good adsorbent, with 100% removal of Congo red in a 15 min saturation time [84]. Other mixed metal oxides for the removal of malachite green oxalate dye were reported by Kumar et al. [85], using ZnO and SiO_2_.The maximum removal rate of Malachite green Oxalate (MGO) was 144.5 mg/g with ZnO, and 149.03 mg/g with SnO_2_. Further studies on dye removal have been tabulated in Table 2.

#### 2.2.3. Metal Oxide Nanomaterials with Antibacterial Activity

An ideal disinfectant to tackle such pathogens should exhibit the following properties, as mentioned by Rutala et al. [94]: (a) Broad antibacterial ability at an ambient temperature over a short period of time, (b) no destructive by-products during and after use, (c) the disinfectant itself should not harm health, (d) economical and has easy applicability, and (e) easy storage, is water soluble, and has safe disposal.

Metal oxides generally execute ideal behaviors as disinfectants. For example [95], Zn/Fe oxide composite nanoparticles as mixed phases of zinc oxide, iron oxide and ferrite phases were tested for their antibacterial activity against *E. coli* and *S. aureus*, where this property depends mainly on the ratio of [Zn/Fe]. When the ratio of the composite was higher, it resulted in better antibacterial properties. Shape-dependent antibacterial effects were observed by Antony et al. [96], where differently shaped copper oxide (CuO) nanomaterials were synthesized by simple sol-gel and hydrothermal approaches, and applied towards Gram positive and Gram negative bacteria. Among the differently shaped CuO crystals, plate-like morphologies showed more effective antibacterial activity than needle or grain shaped morphologies. Similarly, deposits of CuO on cellulose paper, commercial textiles, and poly(ether sulfone) (PES) flat sheets were studied by Booshehri et al. [97]. The main reason for the excellent behavior was due to the release of copper ions from the nanoparticles, and they could be considered as cheap materials to remove pathogens. Metal oxides such as ZnO, Cu_2_O, and NiO supported on natural clinoptilolite were investigated by Jasna et al. [98]. Among the nanoparticles, Cu_2_O and ZnO showed 100% reliability for antibacterial activity against *S. aureus* and *E. coli*, whereas NiO showed a lower affinity towards bacteria. Mixed oxides such as strontium titanate ferrite (STF) were also tested for their antibacterial activity by Zhang et al. [99]. Excellent bactericidal effects were observed against *E. coli* (NiO_5_ CFU/mL) over 15 min in both dark and light conditions, with the reason for this being Sr^2+^ dissociation and the possible contribution of the nanosized properties of STF_0.8_ metal.

### 2.3. Carbonaceous Materials as Pollutant Removal Media

Current trends in purification and separation techniques involve carbon-based materials for many major applications. The surface energy and area, conductivity, thermal stability, and mechanical stability are some of the key factors that enable carbon materials to be dopants or fillers, along with metals or polymers. Enhanced performance was observed with all of the materials when they were coupled with carbonaceous compounds.

#### 2.3.1. Heavy Metal Removal

**Scheme 2:**
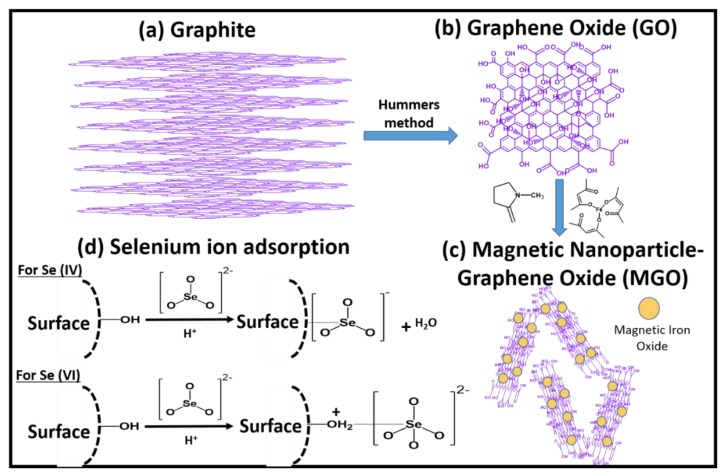
The synthesis of magnetic iron oxide-graphene oxide (MGO). Adopted from Ref. [104].

Magnetic graphene oxide (MGO-Fe_3_O_4_-GO) functionalized with xanthate exhibits ideal behavior as an adsorbent for the removal of Hg(II) with a high capacity of 118.55 mg/g for Hg^2+^ in 180 mm at neutral pH. Thermodynamic results indicated that adsorption was an endothermic and spontaneous process. The Major reason for the high selectivity was due to the specific surface area of graphene and the active xanthate groups [100]. Reduced graphene oxide (RGO)-metal/metal oxide, e.g., MnO_2_/Ag were prepared by simple synthetic methods by Sreeprasad et al. [101], and tested for the removal of Hg(II). Walnut shell-derived activated carbon (AC) was studied in an attempt to remove Hg(II) from industrial liquid streams [102]. AC has shown about 0.45 g/cm^3^ density, 737 mg/g iodine, and 780 m^2^/g Brunauer-Emmett-Teller (BET) surface area as its material property, with a decrease in Hg (II) uptake with an increase in pH. A magnetic graphene oxide (MGO), synthesized by Jin et al., showed a maximum sorption up to 91.29 mg/g in a Cd(II) methylene blue binary system. Tap water samples showed a sorption capacity of 65.39% Cd(II) [103]. Functionalized MGO composites were employed in the removal of selenium ions, which are toxic at concentrations >40 ppb (40 µg/L) (Scheme 2). The presence of MGO (1 g/L) demonstrated a removal efficiency of 80% for Se(VI), and >99.9% for Se(IV) from water at neutral pH [104].

Activated carbon (AC) after chemical and biological treatments (reduced active carbon by sodium sulphite, and modified by baker’s yeast) was studied for the removal of cadmium. The highest sorption capacity was calculated to be 265 µmol/g. A multistage column technique was applied with AC, and it showed a superior rate of cadmium metal recovery [105]. Similarly, silica/AC (2:3) composites exhibited further enhancements when compared to the individual silica material [106].

Surface-treated hydrogen-exfoliated graphene sheets with nitric acid produce hydrophilic functional groups, which activate arsenic (III) & (V) removal quite effectively. The desalination of water, and the removal of sodium, arsenic, and arsenate with a maximum adsorption capacity of 122, 139, and 142 mg/g, were obtained [107]. Other composites such as carbonaceous sulfur-containing chitosan, Fe(III) composites were developed to study the removal of Cu^2+^ from waste water. The majority of sulfur binds with Fe and forms a stable matrix with macropores, which help with trapping most of the Cu^2+^ ions. The Cu^2+^ adsorption was supported by chelation with oxygen moieties and with some sulfur atoms. An adsorption capacity of 413.2 mg/g for Cu^2+^ was observed over 2.5 min, which is considered to be higher than all of the earlier reports for Cu^2+^ removal [108].

**Scheme 3:**
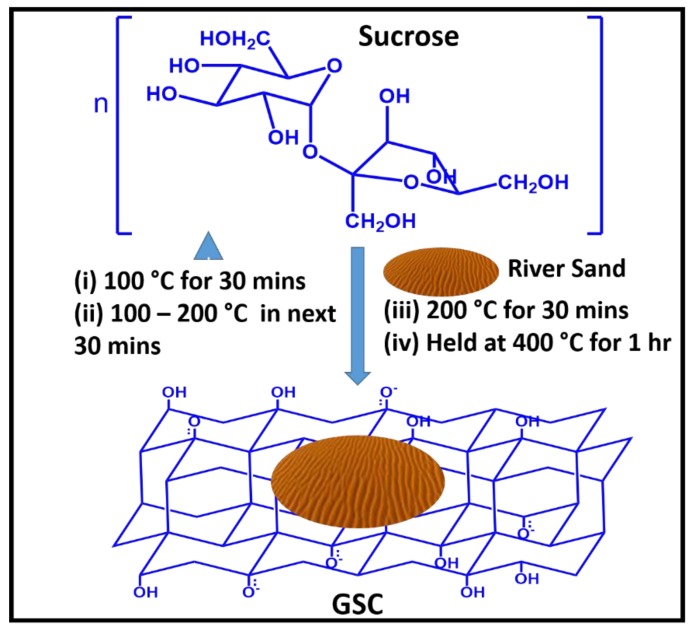
Synthesis of sugar-coated sand. Adopted from Ref. [110].

Graphene magnetic material (Fe_3_O_4_-GS) combinations prepared by Guo et al. [109] showed sorption capacities at 17.29 (Cr(VI)), 27.95 (Pb(II)), 23.03 (Hg(II)), 27.83 (Cd(II)), and 22.0 mg/g (Ni(II)) at a pH range of 3.5. Chelation and ion exchange mechanisms were involved in this removal process. Sand coated with sugar molecules followed by thermal heating for graphitization was reported by Renu et al. [110] for the removal of hexavalent chromium ions (Scheme 3). The increased specific area before and after carbonization helped with the remove Cr(VI) efficiently, and it was found to be 2859.38 mg/g at room temperature, which so far, is an exceptionally high reported value.

Nano-hybrids of magnetic ferrite with graphene oxide nanoparticles has shown adsorption properties for Pb(II), As(III), and As(V). 3D graphene composites showed enhanced properties with the removal of metal ions. So far, many articles were reported on a 3D graphene oxide composite. The synthesis of 3D graphene oxide (GO) hydrogels containing nanoscale-layered double hydroxide (LDH) was reported by Qle et al. [111]. LDH acts as a crosslinking agent between GO nanosheets, and it forms a 3D network, and has been applied for the removal of Cd^2+^ from water with high capability. The major reason for its high capability is due to its hydrophilicity and stability, and the availability of its active sites in aqueous solution. Similarly, a poly(3-amino propyl triethoxysilane) oligomer cross-linked graphene oxide was synthesized by Shenglian et al. [112]. The adsorption capacity of Pb(II) was tested using this 3D network, and it showed an adsorption capacity of 312.5 mg/g at 303 K; the adsorption further increased with increasing temperature. The material showed capability to remove Pb, Cu, and Fe from mixed solutions of metal ions. A direct 3D graphene foam prepared by Yinlin et al. was tested against different metal ions like Zn^2+^, Fe^3+^, Pb^2+^, and Cd^2+^, where the presence of high surface area and oxygen content helps in the efficient removal of the metal ions [113].

#### 2.3.2. Dye Removal

Though polymer materials and metal oxides play a catalytic role in degrading or filtering dye materials, the quenching of highly efficient materials is always anticipated. Carbon-containing mixtures or mixed oxides with carbon are shown to be promising materials for addressing the above issues, to contain the extent of acceptability.

**Scheme 4:**
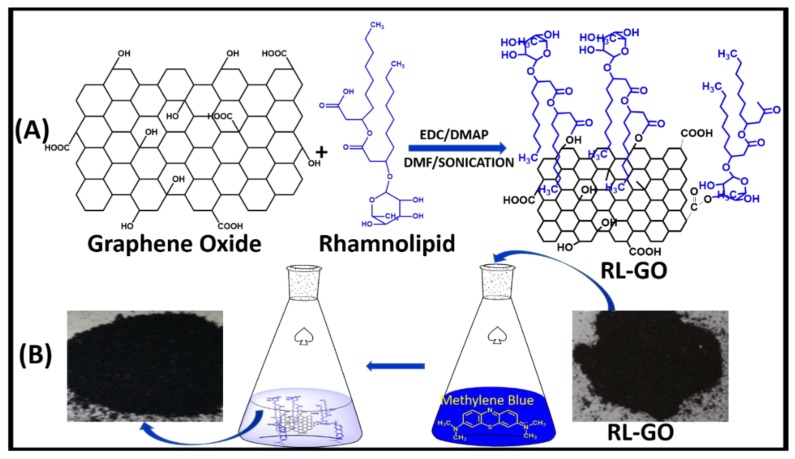
Schematic depiction of the formation of rhamnolipid-functionalized graphene oxide (RL-GO), and its application for the removal of MB. Adopted from Ref. [115]. (**A**) Chemical reaction occurred between GO and RL; (**B**) Pictorial representation of the products.

GO, upon its functionalization with various polymer or organic moieties, opens an extended window for the removal of dye particles. Cui et al. [100] reported a xanthate-functionalized magnetic graphene oxide (Fe_3_O_4_-xGO) in which the MGO removed methylene blue in 120 min at pH 5 in a range of 526.32 mg/g, with a good correlation of kinetics. The major reason for this maximum removal was due to chelation or ion exchange process. Acid yellow dye was tested with AC-Fe_3_O_4_ at pH 5 [114]. Chitosan cross-linked to graphite oxide were tested for black 5 dye, and exhibited a capacity of 277 mg/g at 25 °C. This higher removal capacity was due to ionic interaction/forces between the dye and composite. Similarly, rhamnolipid-functionalized GO by an ultra-sonication method was synthesized by Wu et al. [115]. The mesoporous and functional groups enhances the removal of MB dye with maximum capability (Scheme 4). Through, electrostatic attraction, π–π interactions, and hydrogen bond. MB was further removed in an efficient way by a filtration technique. For example, Robert et al. [116] reported carbon nanofiber for elimination of MB. A high flux (1580 L/m^2^/hr) was reported, which was 10–100 times higher than commercial nano or ultra-filtration membranes. These values were much higher in comparison to carbon nanotubes and granular active carbon. A large surface area, uniformity, and numerous active sites support the enhancement of MB dye adsorption.

#### 2.3.3. Anti-Bacterial Activity

Metal oxide-doped graphene systems exhibit a vast range of anti-bacterial activity. GO was decorated with Ag nanoparticles to form GO-Ag nanocomposites were tested for its antibacterial activity. Ag is well-known for its activity with bacteria, and when GO is coupled with Ag, it enhances its activity further. Microorganism such as *Pseudomonas aeruginosa* do not showed any activity with GO, but when the composite is used, its biocidal activity increases with minimum inhibitory concentration from 2.5 to 5.0 µg/mL. This work by Andreia et al. [117] showed the first direct evidence for the action of GO-Ag, which can inhibit the growth of adhered microbial cells. These materials can also act as bio-coatings. A TiO_2_-RGO composite prepared by the photocatalytic reduction method was applied to *F. solani* spores and *E. coli*. The microorganism’s inactivation rate was much higher than when compared to only TiO_2_. *E. coli* inactivation was observed even after the solar UVA cut-off, which was due to singlet oxygen production with the visible light excitation of TiO_2_-RGO [118]. Carbon nanofiber doped with TiO_2_/ZnO nanoparticles by electrospinning and calcination showed good antibacterial properties. The adsorption characteristics of the carbon nanofiber and the bandgap match with TiO_2_ and ZnO provides the reason for the enhanced properties of the composite [119]. Organic moieties that are coupled with GO also exhibit good antibacterial activity with *B. subtilis* and *C. metallidurans*, with the cell inactivation capacity of 92.3 ± 10% and 99.1 ± 1.3%, respectively. Oxidative stress mechanisms toward the cell cause major bacterial inactivation [120]. The result of zero cytotoxicity with human corneal epithelial cell lines confirms the compatibility of these composites to human exposure.

## 3. Oil-Water Separation and Removal using Nanofiber Membranes and Carbonaceous Materials

**Figure 2:**
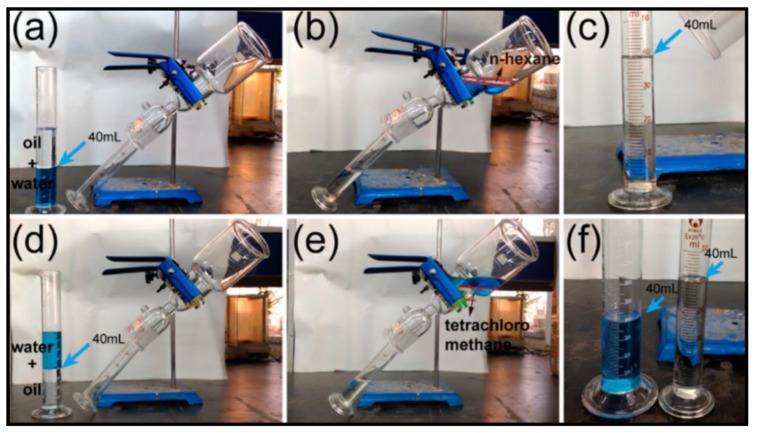
Photographs of the oil-water separation process: (**a**) a membrane fixed between a glass tube and a conical flask with a tilt angle of 45°, (**b**) a mixture of *n*-hexane (40 mL) and water (40 mL) poured into an upper glass tube and *n*-hexane passing through the membrane but water repelled, (**c**) hexane collected in a measuring cylinder after separation, (**d**–**f**) a similar process for separating tetrachloromethane (heavier than water) and water (water dyed with methyl blue). Adopted from Ref. [122].

Novel polysulphone electrospun nanofiber mats were used as effective membranes for oil-water separation by Obaid et al. [121]. Surface modification was done by the insertion of NaOH nanoparticles, and the formation of thin polyamide polymers. The insertion of thin layers decreases the contact angle, and shows a good degree of efficiency with oil-water separation at a relatively high water flux of 5.5 m^3^/m^2^ day. Similar studies was conducted by Caili et al., using PIM-1/polyhedral oligomeric silsesquioxane (POSS). The membrane showed superhydrophobic-superoleophilic properties when the POSS concentration increased to 40%. The membrane showed a tremendous degree of efficiency towards the oil-water mixture of up to 99.95%, and it extended the separation of water-in-oil emulsions with greater efficacy (99.97%) (Figure 2). This membrane was also subjected toward dye absorption from oils, and it exhibited an adsorption capacity of about 8.33 mg/g for Blue 35, and 7.49 mg/g for Oil Red [122]. When the microfiber was dip-coated with fluorinated alkylsilane and PIM-1, the membrane surface was modified to show a superhydrophobic surface, and resulting in the successful separation of oil and water, with a maximum separation capacity of 99.96% [123].

Similarly, the immersion of a PVDF membrane into aqueous dopamine solution for a day showed stable superoleophobicity in sea water, with an oil contact angle of 152 ± 0.3°. The membrane showed high oil-water separation, and high permeability [124]. Conventional UF membranes were compared with TFC membranes by Mehrdad et al. Efficient flux and rejection of both membranes were identified, and they showed that the TFC’s pure flux rose from 20% to 160%, in comparison to Polysulfone (PSF) asymmetric membrane [125]. Poly(vinylidenefluoride-co-hexafluoropropylene) (PVDF-Co-HFP) nanofibers modified with cellulose were studied for oil-water separation by Farah et al. [126]. Great control of porosity and wettability were observed for the 3D-impregnated cellulose, and at 15 wt % cellulose, the modulus and the tensile strength increased from 17 MPa to 54 MPa, and from 5.5 mPa to 8.6 mPa, respectively. It showed about 99.98% oil-water separation efficiency.

**Scheme 5:**
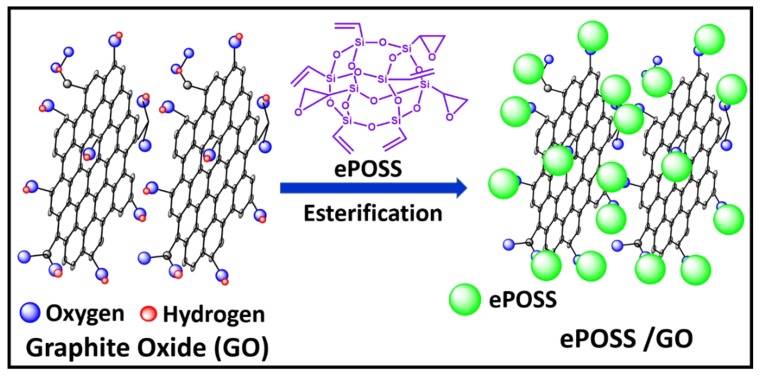
Esterification of GO with ePOSS Adopted from Ref. [128].

Graphene/CNT aerogels with 3D interconnects were reported for efficient oil-water removal. The reduction of GO and acid-treated CNT by ferrous ions was employed, and it exhibited outstanding performance for the removal of petroleum products, fats, and organic solvents. The aerogel showed the capacity for removing 28 L of oil/g [127]. GOs functionalized with ePOSS (epoxy-functionalized polyhedral oligomeric silsesquioxane) with a strong super hydrophobic (contact angle of ~145°) nature were tested over the oil-water separation (Scheme 5) [128].

Another report on Al_2_O_3_-modified GO to address the high permeate flux and oil rejection in oil/water emulsions was published by Xuebing et al. [129]. GOs were coated on Al_2_O_3_ membranes homogenously, therefore resulting in a covalent bond formation between the membrane and GO. A high flux rate of 522 and 667 L/m^2^/hr was observed, and this composite showed good tolerance towards effective oil water removal. Janus polymer/CNT membranes were prepared by grafting hydrophobic polystyrene and hydrophilic *N*,*N*-dimethylaminoethylethacrylate (PDMAEMA) with photoactive CNT membranes. Surfactant-stabilized water in oil, and oil in water emulsions were effectively separated by these membranes, due to anisotropic wettability [130].

## 4. Conclusions

Nanotechnology assists in devising many solutions for environmental issues that are considered life-threatening for current and future generations. Materials with high surface area has shown excellent surface properties, and exhibit tremendous efficiencies with the individual and composite materials towards solving problems. Further developments are still an ongoing process at the research level, and they focus more on addressing more complicated issues pertaining to water purification. These nanocomposites show multiple aspects for the removal of pollutants from water, and they poses positive signs for further developments. Easy techniques with more enhanced nanomaterial property will exhibit more promise for use in water separation and purification. Recent trends in 2D and 3D materials create many pathways for careful engineering of materials for target pollutants. The chemical or physical modification of these 2D materials will contribute more towards the processes and the resources for cleaning water.

## Figures and Tables

**Table 1:** Composite nanofiber membranes for heavy metal removal.

Nanofiber Material	Target Pollutant	Pollutant Removal Efficiency	Method	pH	Ref.
Citric acid-modified acrylic fibers	Cu(II) and Ni(II)	Ni(II) = 2.55 mg/g at 250 mmol/g Cu(II) = 2.56 mg/g at 250 mmol/g	Grafting, Electrospinning	6	[46]
Polyacrylonitrile-polypyrrole (PAN/PPy) core/shell nanofibers	Cr (VI)	28.5 mg/g at 100 mg/L in Cl^−^	Electrospinning, Adsorption	2–5	[34]
Chitin nanofibrils	Pb(II), Ni(II),Cd(II), Cu(II)	303.49 mg/g, 134.72 mg/g 330.15 mg/g, 141.08 mg/g	Mechanical defibrillation	5	[47]
Poly(vinyl alcohol) (PVA)/NaX nanozeolite	Ni(II) and Cd(II)	342.8 and 838.7 (mg/g)	Electrospinning	5	[48]
Phosphorylated PAN-based nanofiber (P-PAN) mat	Ag(I), and Cd(II), Pb(II), Cu(II)	102.40 mg/g, and 18.89 mg/g, 98.06 mg/g, 78.03 mg/g	Electrospinning and chemical modification	6	[49]
Silk Fibroin/cellulose acetate-blend nanofibers	Cu(II)	22.8 mg/g	Electrospinning	-	[50]
Nanofibers with sodium titanate (TNF-A), potassium/sodium titanate (TNF-B), potassium titanate (TNF-C)	Ba(II) (as substitute of 226Ra(II)) and Pb(II)	Ba(II)^+^—1.93 mmol (TFN-A), 1.67 (TFN-B), 1.74 (TFN-C) Pb(II)—1.91 mmol (TFN-A), 1.88 (TFN-B), 1.79 (TFN-C)	Chemical approaches	6–7	[51]
Cysteine-modified polypyrrole propylic acid nanofibers	Cr(III)	1.75 g Cr/g of polymer	Electrochemical template-directed method	3.28	[42]
Iron oxide–alumina mixed nanocomposite fibers using Polyvinylpyrrolidone (PVP)	Ni(II), Hg(II), Cu(II), Pb(II)	32.36 mg/g, 63.69 mg/g, 4.98 mg/g, 23.75 mg/g	Electrospinning, Sol gel	6	[52]
Sodium titanate nanofibers	(a) Ni(II), Zn(II), Cu(II), and Cd(II) (b) Pb(II)	(a) 60, 83, 115, and 149 mg/g (b) 250 mg/g	Hydrothermal	(a)6.5 (b) 4	[53]
Rhodanine and poly(methyl methacrylate)	Ag(I) and Pb(II)	140 and 115 mg/m^2^	Electrospinning	-	[40]

**Table 2:** Metal oxides in dye degradation and/or removal.

S. No	Metal Oxide	Target Dye	Removal Efficiency	pH	References
1	Cobalt (Co) and nickel (Ni) oxide (Co_0.4_Ni_0.4_O_0.2_)	Methylene blue (MB) and Procion red (PR)	MB—20% PR—70%	9.5	[86]
2	Pectin-thorium (IV) tungstomolybdate (Pc/TWM)	Methylene Blue	76%	-	[87]
3	Horseradish peroxidase (HRP) was encapsulated in phospholipid-template titania particles	Direct Black-38	79.72%	6–9	[88]
4	Fe_3_O_4_-ZnO magnetic semiconductor nanocomposite	Methylene Blue, Methyl Orange, Rhodamine B	-	Photo degradation	[89]
5	Magnetic Fe_3_O_4_/ZnCr-LDH (Layered double hydroxide)	Methyl Orange (MO)	81.23%	6.4–7.3	[90]
6.	BiFeO_3_/a-Fe_2_O_3_ core/shell composite	MO	80%	5.2	[91]
7	Fe-Al binary oxide (5:5)	AB25, AO7, MB	100%, 98%, 88%	-	[92]
8	Chromium spheres	Congo Red	75%	-	[93]
